# Differential Expression of mRNA in Peripheral Blood Mononuclear Cells May Predict Postoperative Atrial Fibrillation in Coronary Artery Bypass Surgery Patients

**DOI:** 10.1007/s12265-024-10524-8

**Published:** 2024-07-15

**Authors:** Mingqi Tan, Xiankun Liu, Lianqun Wang, Nan Jiang, Yunpeng Bai, Zhigang Guo

**Affiliations:** 1 Academy of Medical Engineering and Translational Medicine, Tianjin University, Tianjin, China; 2 Department of Cardiac Surgery, Chest Hospital, Tianjin University, Tianjin, China; 3 Tianjin Institute of Cardiovascular Diseases, Chest Hospital, Tianjin University, Tianjin, China; 4 Clinical School of Thoracic, Tianjin Medical University, Tianjin, China; 5 Tianjin Key Laboratory of Cardiovascular Emergency and Critical Care, Tianjin Municipal Science and Technology Bureau, Tianjin, China

**Keywords:** Postoperative Atrial Fibrillation, Coronary Artery Bypass Grafting, LASSO Predictive Model

## Abstract

**Abstract:**

Postoperative Atrial Fibrillation (POAF) frequently follows Coronary Artery Bypass Grafting (CABG) surgery. This prospective study investigates genes linked to POAF in CABG patients, aiming to create a predictive model. Employing differential gene and methylation analyses, the study identified four genes (WARS2, CKAP2, CHI3L1, HSD17B6) associated with POAF. Preoperative plasma samples and clinical data were collected from 139 CABG patients, categorized into POAF (+) (43) and POAF (-) (96). Real-time quantitative PCR assessed gene expression, and a predictive model using the LASSO method demonstrated robust performance, with AUC values of 0.8895 in the training set and 0.7840 in the test set. This pioneering study integrates genomics and clinical data, suggesting WARS2, CKAP2, and CHI3L1 as potential indicators for POAF prediction.

**Graphical Abstract:**

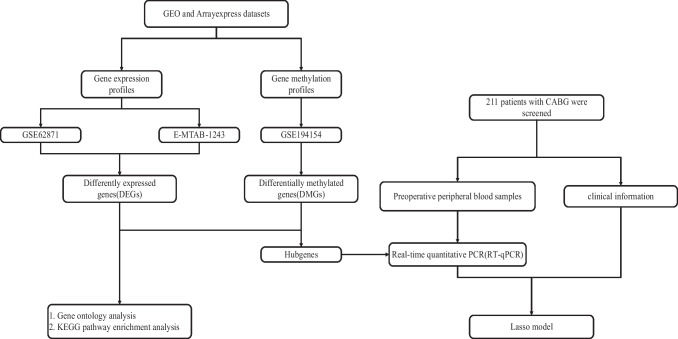

**Supplementary Information:**

The online version contains supplementary material available at 10.1007/s12265-024-10524-8.

## Introduction

The onset of postoperative atrial fibrillation (POAF) following coronary artery bypass grafting (CABG) is a common occurrence. After undergoing CABG surgery, approximately 20% to 40% of patients may experience POAF [1]. In the past, for generally healthy patients undergoing CABG, POAF was often considered a symptom that had minimal impact on the body and was easily manageable. There has been insufficient attention given to POAF because it causes minimal direct harm to patients. However, recent research indicates an association between POAF and an increased risk of long-term death, the long-term risk for thromboembolic events [2], and stroke [3]. The research indicates that patients with POAF undergoing CABG surgery have a significantly higher long-term mortality rate [4]. The probability of experiencing a stroke was three times higher in patients with (+) POAF compared to patients without (-) POAF [5]. Additionally, POAF patients were more prone to recurrent atrial fibrillation (AF) in subsequent episodes. POAF also contributes to increased treatment costs for patients, as those who experience POAF tend to have longer stays in the Intensive Care Unit, leading to higher treatment expenses [6].

In current research on predicting POAF, most studies analyze preoperative clinical information from patients to identify potential risks for early prediction of POAF. Additionally, some studies [7] approach the prediction of POAF from the perspective of transcriptomics, investigating whether there are specific differences in the expression of genes between POAF (+) and POAF (-) patients. This exploration aims to understand the exact distinctions and utilize them for predicting the occurrence of POAF [8]. Recent investigations have explored the predictive value of preoperative peripheral blood microRNA expression levels, circRNA expression levels, plasma proteomics, and metabolomics in anticipating POAF in patients [9–11]. Despite these advances, there remains a paucity of research on predicting POAF by integrating gene expression in patients’ peripheral blood with clinical data. Leveraging bioinformatics methodologies and the public datasets from Gene Expression Omnibus (GEO) and ArrayExpress, this study aims to identify differential genes between POAF (+) patients and POAF (-) patients undergoing CABG surgery, with the expectation that these differential genes may enhance the prediction of POAF. Based on these inquiries, the hypothesis is formulated that preoperative peripheral blood gene expression in patients holds potential utility in predicting POAF. This study is designed to scrutinize and substantiate this hypothesis.

## Methods

### Data Collection

**Fig. 1:**
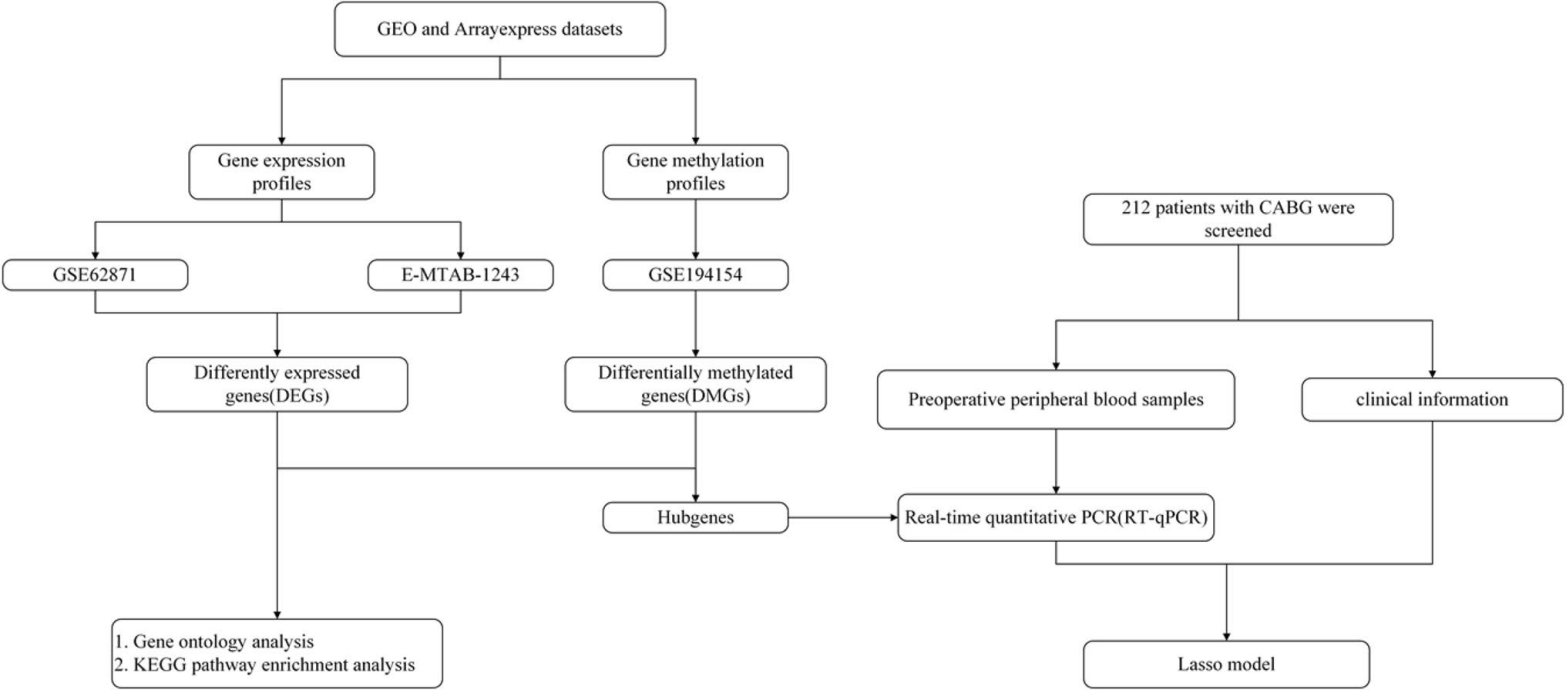
Flow chart of preparation, processing, and analysis

The methodological framework for data analysis in this investigation is depicted in Fig. 1. This study utilized raw data from dataset GSE62871, comprising 9 right atrial tissue samples procured from patients exhibiting sinus rhythm (SR) and 7 samples from patients with POAF. Additionally, DNA methylation profiles were obtained from dataset GSE194154, which includes 63 pre-operative whole blood samples from individuals with SR and 47 from those with POAF. Both datasets were retrieved from GEO (https://www.ncbi.nlm.nih.gov/geo/). Furthermore, dataset E-MTAB-1243 was acquired, consisting of 10 heart tissue samples from SR patients and 6 from those with POAF, downloaded from the ArrayExpress database (https://www.ebi.ac.uk/arrayexpress/). These comprehensive datasets formed the empirical basis for the subsequent analytical processes.

### Data Pre-Processing and Screening

Log_2_-transformation and normalization of the expression profiles for datasets GSE62871 and E-MTAB-1243 were executed utilizing the voom methodology within the R package ‘limma’. Subsequently, probe identifiers were mapped to gene symbols using the corresponding annotation files. Genes registering expression counts below the threshold of 10 in excess of 25% of the samples were excluded from further analysis.

In regards to dataset GSE194154, inclusion criteria were stringently applied as follows: i) exclusion of patients subjected to composite surgical procedures; ii) exclusion of patients with a history of tobacco use; iii) exclusion of patients diagnosed with diabetes. The initial cohort of 110 samples was thus curtailed to 36 samples post-screening. This refined subset comprised data from 12 POAF (+) patients and 24 POAF (-) patients.

### Identification of Differentially Expressed Genes (DEGs)

Two datasets, GSE62871 and E-MTAB-1243, were leveraged to pinpoint DEGs. Differential expression analysis was performed using the ‘limma’ package in R. For GSE62871, the criteria for significance were set as |log2 Fold Change (FC)| > 0.585 and *P* < 0.05. Genes with log2 FC > 0.585 and *P* < 0.05 were designated as highly expressed in POAF, while those with log2 FC < -0.585 and *P* < 0.05 were considered lowly expressed in POAF. The same criteria were applied to E-MTAB-1243 to identify DEGs. Additionally, DEGs were visually represented through a volcano plot, crafted using the ‘ggplot2’ package.

### Identification of Differentially DNA-Methylation Genes (DMGs)

The selected sample data from GSE194154 was imported using the function methRead from the R package ‘methylKit’. Following the import, the samples were filtered based on sequencing depth using the function filterByCoverage. The function calculateDiffMeth is used to calculate the degree of methylation at each site and convert it into percentiles. Differential methylation positions (DMPs) were selected based on a q-value < 0.01 and a methylation percentage difference greater than 20%. Additionally, all DMPs were visually through a volcano plot. The R package ‘genomation’ is employed to annotate the obtained DMPs, yielding the corresponding differentially methylated genes.

### Function Enrichment Analysis

Gene ontology (GO) pathway enrichment analysis of the DEGs from GSE62871 was conducted utilizing the ‘ClusterProfiler’ package in R. Significance was ascribed to pathways with a p-value < 0.05. Additionally, KOBAS 3.0 [12] (http://bioinfo.org/kobas) facilitated the statistical enrichment assessment of DEGs within the Kyoto Encyclopedia of Genes and Genomes (KEGG) pathways, with significance again determined by a p-value < 0.05. The results of GO and KEGG were visualized with the aid of the ‘ggplot2’ package in R.

### Patient Selection

Preoperative peripheral blood samples were collected from patients designated for CABG at the Tianjin Chest Hospital (Tianjin, China). The Ethics Committee of the Tianjin Chest Hospital authorized the study with regulatory and ethical permission (2022YS-032-1).

Participant selection was predicated on established inclusion criteria: i) Candidates were to undergo their initial CABG operation for coronary heart disease exclusively; ii) Absence of preoperative ventricular arrhythmias was confirmed for all patients; iii) None of the patients had suffered an acute myocardial infarction prior to the surgical intervention; iv) Congenital heart diseases were not present among the patient cohort.

POAF was defined as multiple AF lasting >30 seconds, recorded by electrocardiogram monitor or continuous wireless rhythmic monitoring, began immediately after surgery or later before discharge, requiring anti-AF treatment (usually intravenous amiodarone). The electrocardiographic data were stored and analyzed after discharge. These assignments were made solely on the basis of the presence or absence of POAF.

### Data Collection and Definition of Variables

Demographic and clinical parameters were systematically collated for analysis, encompassing sex, age, body mass index (BMI), diabetes mellitus (DM), and hypertensive condition. Additionally, preoperative evaluations were recorded, including hemoglobin (Hb) levels, serum creatinine (Scr) concentrations, and left ventricular ejection fraction (LVEF) measurements. Information on the preoperative administration of heart rate control medications (Drug Use) and surgical details (duration of surgery, incidence of right coronary artery bypass grafting (RCAB), and the extent of single or multiple arterial bypass grafting) was also accrued. The principal endpoint of this investigation was the occurrence of POAF. Thus, the study cohort was composed of patients with no prior history of AF, no manifestations of any AF type preoperatively, and who presented with new-onset AF post-surgery.

### mRNA Isolation and Real-Time Quantitative PCR (RT-qPCR)

Total RNA from peripheral blood mononuclear cells (PBMCs) was extracted using the RNAprep Pure Blood Kit (DP443, TIANGEN, Beijing, China) following the manufacturer’s protocol. Quantitative gene expression analysis via reverse transcription-quantitative PCR were performed to validate the hubgenes expression. The total RNA extracted from PBMCs was reverse transcribed into cDNA using Hifair® III 1st Strand cDNA Synthesis SuperMix for qPCR (gDNA digester plus) kit (11141ES, YEASEN, Shanghai, China). The steps were 42°C for 2 min, 25°C for 5 min, 55°C for 15 min and 85°C for 5 min to end the reaction.

The RT-qPCR experiments were performed using Hieff UNICON® Universal Blue qPCR Master Mix kit (11184ES, YEASEN, Shanghai, China) in 7500 Real-Time PCR system (Applied Biosystems, San Francisco, CA, USA). The following thermocycling conditions were applied: 95°C for 2 min; followed by 40 cycles at 95°C for 10 sec, 60°C for 32 sec; 95°C for 15 sec, 60°C for 60 sec,95°C 15 sec and 60°C 15 sec for final extension. Standard and melting curves were generated in every plate for each gene to ensure that the reaction is efficient and specific. The cycle threshold value of β-actin acted as the internal control. The relative expression levels of different genes were analyzed via the 2^−ΔΔCT^ method.

The primer sequences were acquired from PrimerBank (http://pga.mgh.harvard.edu/primerbank), and the specific primer sequences are delineated in Online Resource 1.

### Least Absolute Shrinkage and Selection Operator (LASSO) Model

Before constructing the LASSO model, we performed preprocessing on the data. Firstly, we utilized the R package ‘mice’ to perform multiple imputations on the dataset, addressing missing values. Subsequently, we standardized the data. For variables such as BMI, LVEF, Hb and Scr, we applied quartile categorization. Age was binarized using 65 years as the threshold, and for operation time, we employed a binary classification with the median value as the threshold.

To identify factors associated with POAF prediction, we conducted a LASSO regression analysis using 5-fold cross-validation to select Lambda values. We divided the dataset into training and testing sets in an 8:2 ratio to construct and evaluate the LASSO model. Subsequently, a multivariate logistic regression analysis was performed, including factors selected from the LASSO regression analysis. The area under the curve (AUC) of receiver operating characteristic (ROC) was utilized to determine the effectiveness of features in discriminating POAF (+) samples from POAF (-) samples and performed by using the ‘pROC’ package.

### Statistical Analysis

All statistical analyses were conducted using R (version 4.1.2). Group comparisons were undertaken for continuous variables using Student’s t-test for normally distributed variables. The chi-square test was utilized to examine categorical variables. LASSO regression analysis was carried out using the ‘glmnet’ package in R. The p-value corresponding to the AUC of the ROC curve was computed using IBM SPSS Statistics 25 software. All statistical analyses were two-sided with *P* < 0.05 were regarded statistically significant.

## Results

### DEGs Between POAF (+) and POAF (-) Samples

**Fig. 2:**
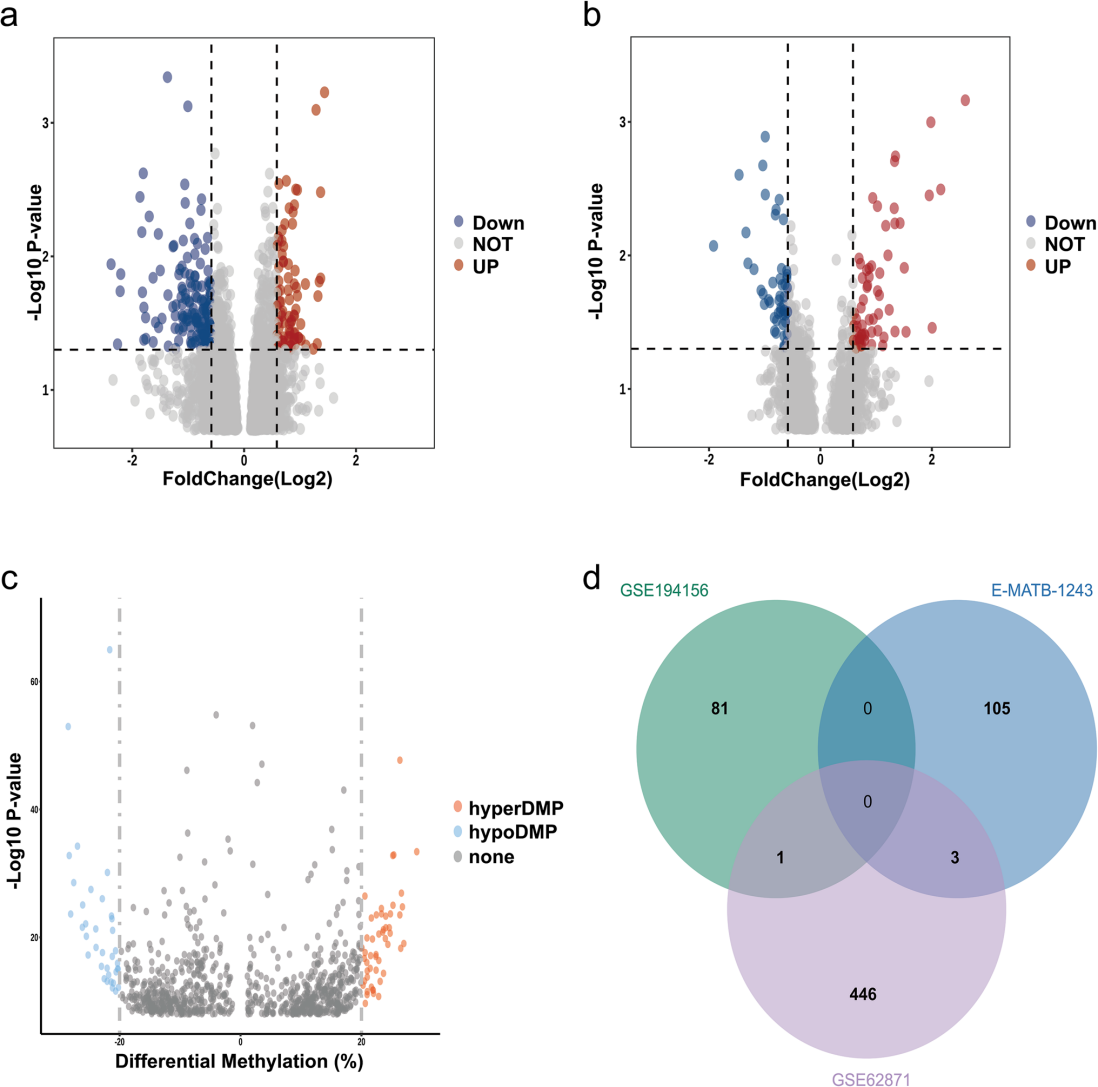
identification of DEGs and DMGs between POAF (+) and POAF (-) samples. Volcano plots of the differential gene expression data from GSE62871 (**a**) and E-MTAB-1243 (**b**). In the volcano plots, the red points show up-regulated genes (log2 FC > 0.585 and *P* < 0.05 in GSE62871; log2 FC > 0.585 and *P* < 0.05 in E-MTAB-1243), whereas the blue points represent down-regulated genes (log2 FC < -0.585 and *P* < 0.05 in GSE62871; log2 FC < -0.585 and *P* < 0.05 in E-MTAB-1243). (**C**) Volcano plots of the differentially methylated positions from GSE194154. (**D**) The overlapped genes between DEGs and DMGs

After pre-processing of the dataset GSE62871 and E-MTAB-1243, DEGs were screened out, according to the value of *P* and |log2 FC|. In GSE62871, DEGs were identified if the cutoff value met the criterion of |log2 FC| > 0.585 and *P* < 0.05. In E-MTAB-1243, DEGs were identified if the cutoff value met the criterion of |log2 FC| > 0.585 and *P* < 0.05. The volcanic diagram for all genes was shown in Fig. 2. We identified 252 up-regulated and 198 down-regulated genes in GSE62871 (Fig. 2a); 60 up-regulated and 48 down-regulated genes in E-MTAB-1243 (Fig. 2b).

### DMGs Between POAF (+) and POAF (-) Samples

Through comparison between the data of patients with POAF (+) and POAF (-), we identified 93 DMPs, with 54 being hypermethylated and 39 being hypomethylated. After annotating these DMPs, we obtained 82 DMGs, of which 52 were hypermethylated and 30 were hypomethylated. The volcanic diagram for all genes was shown in Fig. 2c.

Additionally, we dropped out the duplicated genes among DEGs and DMGs (Fig. 2d). The Venn diagram was drawn online using the website jvenn (http://bioinfo.genotoul.fr/jvenn) [13]. The gene WARS2 was overlapped from DEGs from GSE62871 and DMGs from GSE194154. The gene CKAP2, HSD17B6, CHI3L1 were overlapped from DEGs from GSE62871 and DEGs from E-MTAB-1243. The detailed information regarding the DEGs and DMGs can be found in Online Resource 2.

### Enrichment Analysis of DEGs

**Fig. 3:**
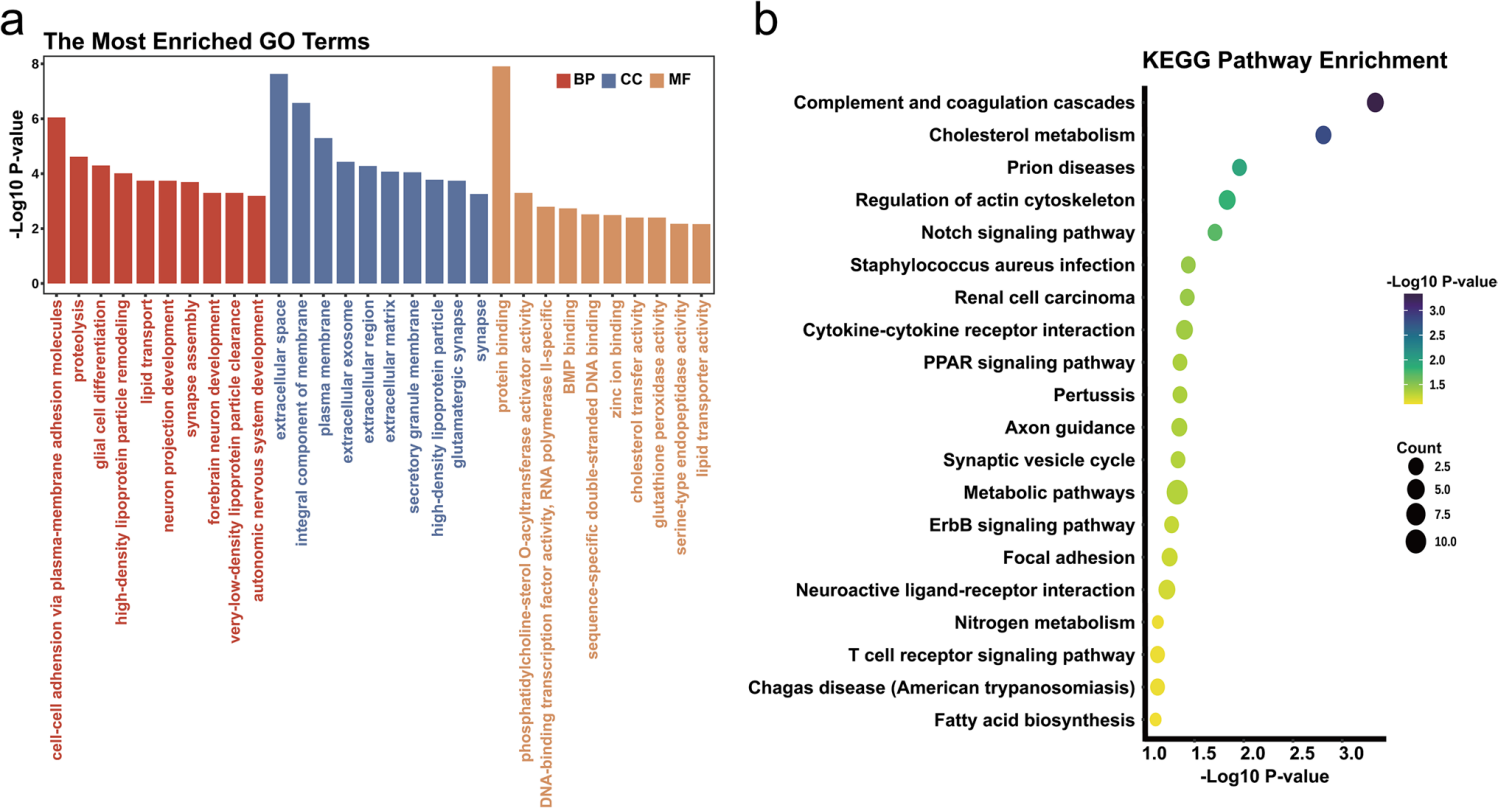
The Significant enriched Gene Ontology terms and KEGG pathways of DEGs from GSE62871. (**a**) The results of Gene Ontology enrichment analysis were presented separately for the top 10 results in BP, CC and MF. (**b**) The top 20 results of KEGG pathway enrichment analysis were displayed in terms of relevance

Functional enrichment analysis on the DEGs from GSE62871 was performed, based on GO and KEGG databases. As shown in Fig. 3a, the ontology composed of three domains (Biological Process (BP), Cellular Component (CC) and Molecular Function (MF)). The enriched biological processes were mainly involved in cell-cell adhesion via plasma-membrane adhesion molecules, synapse assembly, proteolysis, high-density lipoprotein particle remodeling. The cellular components were mainly enriched in extracellular space, whereas the enriched molecular functions were mainly involved in protein binding. KEGG pathway analysis was shown in Fig. 3b. The Complement and coagulation cascades were the most enriched pathways, followed by cholesterol metabolism, prion diseases, regulation of actin cytoskeleton and notch signaling pathway, et al.

### Patient Selection and Peripheral Blood Collection

From April 2023 to September 2023, preoperative plasma samples from isolated first-time CABG patients were prospectively collected and frozen. During this period, there were totally 211 patients undergoing CABG. There were 139 patients who were eligible for the study. After CABG, there were 43 POAF (+) patients and 96 POAF (-) patients. The collected patient clinical data were shown in Table 1.

**Table 1:** Comparison of baseline characteristics between POAF (+) and POAF (-) patients

Variables	POAF (+) (*N*=43)	POAF (-) (*N*=96)	p-value
Sex (male)	34 (79.1%)	69 (71.9%)	0.493
Age (years)	69.0 ± 7.65	66.5 ± 8.23	0.194
DM	22 (51.2%)	39 (40.6%)	0.331
BMI (Kg/m^2^)	25.4 ± 3.05	25.3 ± 3.40	0.862
Hypertension	27 (62.8%)	66 (68.8%)	0.62
Hb (g/L)	138 ± 14.8	131 ± 13.6	0.0084
Scr (umol/L)	80.0 ± 17.2	77.8 ± 21.7	0.523
LVEF (%)	57.8 ± 7.13	58.7 ± 6.33	0.481
Drug Use	31 (72.1%)	76 (79.2%)	0.485
Operation Time (min)	215 ± 68.6	215 ± 52.9	0.99
RCAB	24 (55.8%)	64 (66.7%)	0.3
Multiple Bypass	37 (86.0%)	89 (92.7%)	0.351

Values are presented as n (%), mean ± standard deviation. *DM* diabetic mellitus, *BMI* body mass index, *Hb* Hemoglobin, *Scr* serum creatinine, *LVEF* left ventricular ejection fraction, Drug Use: preoperative heart rate medication, RCAB: incidence of right coronary artery bypass grafting

### Validation of Gene Expression by RT-qPCR

**Fig. 4:**
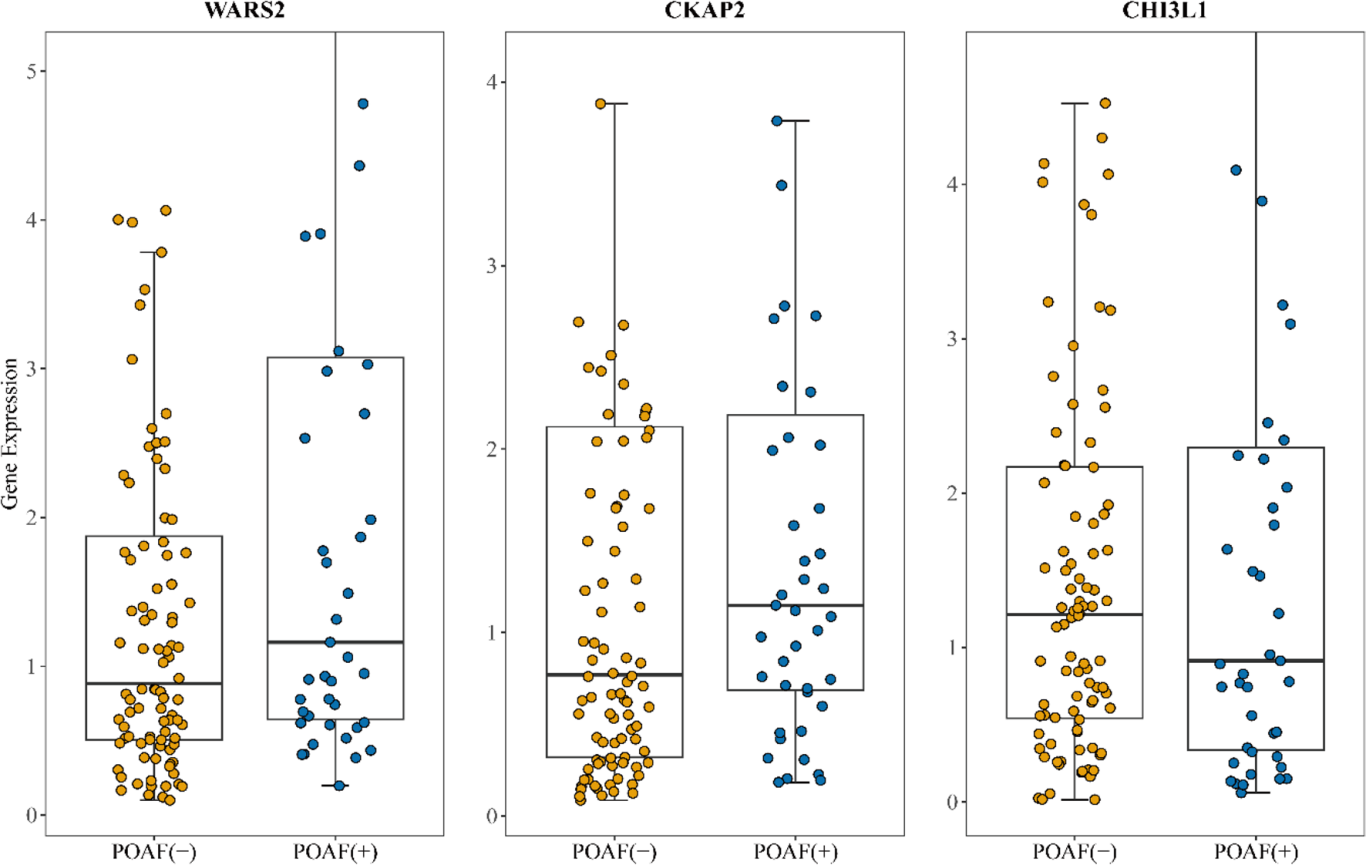
Gene expression in patient peripheral blood detected by RT-qPCR

The comparison of gene expression between POAF (-) and POAF (+) groups yielded the following results: WARS2 (0.883 ± 4.29 vs 1.16 ± 38.3; *P* = 0.16), CKAP2 (0.914 ± 3.89 vs 1.21 ± 13.3; *P* = 0.571), and CHI3L1 (1.15 ± 18.7 vs 0.770 ± 36.5; *P* = 0.792). Notably, the comparison results for these three genes did not reach statistical significance. The gene expression levels were visually depicted using boxplots in Fig. 4.

### Screening Predictive Factors Using LASSO Logistic Regression Analysis

**Fig. 5:**
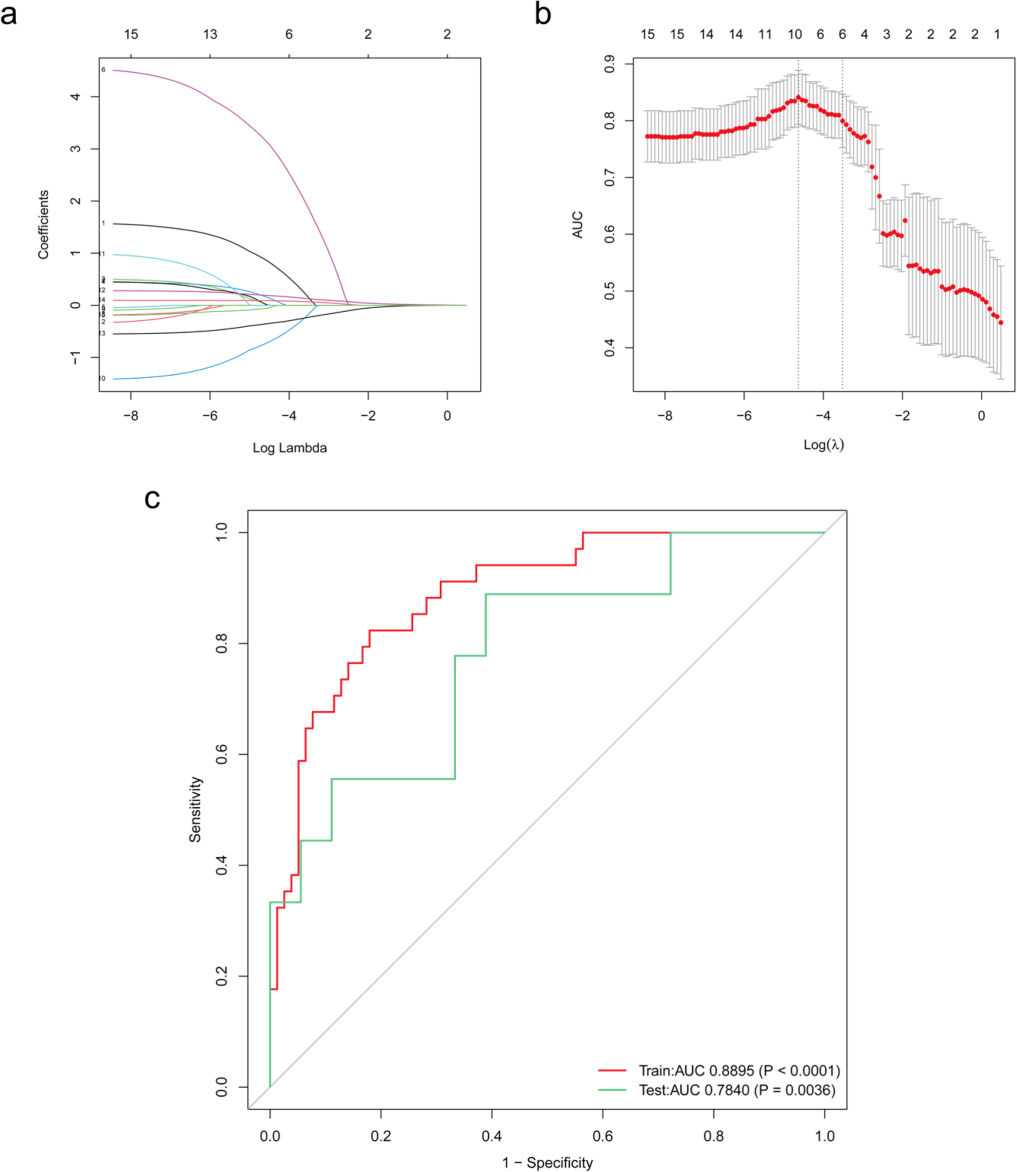
LASSO model construction and ROC curve chart. (**a**) Coefficient profiles for 15 characteristics were generated from logarithmic sequences using LASSO regression analysis. (**b**) Cross-validation was used to select the optimal parameter (λ) for the LASSO model. The binomial deviation of partial likelihood deviation curves is shown versus log_10_(λ). The dotted vertical lines indicate the best values of 1SE (1-SE criterion) using the minimum and maximum criteria. (**c**) ROC curves of LASSO model on training set and test set

LASSO regression analysis and cross-validation were performed for each influencing factor, and the independent variables were further screened. The value with the smallest verification error (log_10_λ = -4.629) was selected to fit the regression model, and there were 10 variables of the model in total (Fig. 5), including RCAB, DM, LVEF, preoperative Hb, BMI, age, Scr, WARS2, CKAP2, CHI3L1.

$$Risk Score = 3.188*(Hb) + 0.889*(age) + 0.193*(WARS2) + 0.173*(DM) + 0.090*(CKAP2) + 0.038*(BMI) + 0.032*(Scr) - 0.063*(LVEF) - 0.361*(CHI3L1) - 0.745*(RCAB).$$

## Discussion

Although the potential harm of POAF to patients has not received sufficient attention in the past, recent research indicates a significant association between the occurrence of POAF and both short-term and long-term patient outcomes [2]. Specifically, a clear relationship has been established between long-term mortality, ischemic stroke, and POAF. Furthermore, potential associations exist between POAF and the recurrence of atrial fibrillation, heart failure, and vascular embolic events [14].

The advancements in predicting POAF have been bolstered by developments in clinical data analysis and genomic research. An array of risk factors has been correlated with POAF, including demographic indicators such as age [15] and sex, anthropometric measures like BMI [11], clinical parameters such as hypertension [16], biochemical indices including Scr [16], cardiac performance metrics like LVEF [17] and medical antecedents encompassing DM, a history of congestive heart failure [18], chronic obstructive pulmonary disease [18], non-valvular heart disease [18], pump or off-pump surgery [19], and obstructive sleep apnea [20]. The etiology of POAF is recognized as being complex and multifactorial, with the precise mechanisms still to be fully determined.

Research into miRNA expression has yielded promising predictive insights for POAF. For instance, studies such as those conducted by Harling [21] have revealed differential miRNA expression in the right atrial appendage of patients, indicating the potential of these miRNAs as predictive biomarkers for POAF. As pathological processes progress, affected organs or tissues may release distinct proteins that potentially act as biomarkers for the condition. Li’s research [9] predicted the onset of POAF by analyzing unique protein profiles and metabolic variations observed in the peripheral blood samples of patients suffering from POAF. The premise of the research delineated in this paper is predicated on the understanding that modifications in gene expression are precursors to consequent changes in protein secretion.

Based on existing research and the current hospital medical records system, we have chosen clinically relevant indicators that have been proven to be associated with or have a correlation with POAF. These indicators include age, gender, DM, BMI, Scr, LVEF, hypertension, and Hb level. Despite research in genomics and clinical information for predicting POAF, studies combining both aspects for POAF prediction are still relatively scarce. Therefore, the study endeavors to employ bioinformatics techniques to identify genes linked to POAF and amalgamate these findings with recognized risk factors, thereby devising a more robust predictive model for POAF.

In this research study, we utilized the GEO public database for bioinformatics analysis to gain a comprehensive understanding of gene expression changes in patients with POAF. Differential expression analysis of the datasets GSE62871 and E-MTAB-1243 revealed 252 upregulated genes and 198 downregulated genes in GSE62871, along with 60 upregulated genes and 48 downregulated genes in E-MTAB-1243. Furthermore, differential methylation analysis of the dataset GSE194154 identified 54 hypermethylated sites and 39 hypomethylated sites, resulting in 52 hypermethylated genes and 30 hypomethylated genes after annotation. Visualization of the intersection of the differentially expressed genes and differentially methylated genes through a Venn diagram highlighted four genes (WARS2, CKAP2, CHI3L1, HSD17B6) that were common between the two sets of genes.

In our study, we took a pioneering approach by integrating genomics with clinical data to construct a predictive model for POAF in patients undergoing CABG. We collected preoperative peripheral blood samples from patients to assess the expression levels of genes identified earlier through bioinformatics methods. This genomic data was combined with preoperative, intraoperative physiological data, and surgery-related information already collected to construct a comprehensive predictive model for POAF. In constructing the LASSO model, we partitioned the data into training and testing sets in an 8:2 ratio. To evaluate the predictive performance of the model, we generated ROC curves. The results revealed an AUC value of 0.8895 for the training set and 0.7840 for the testing set, indicating a robust predictive performance. In the LASSO model, WARS2, CHI3L1, and CKAP2 were retained as features, suggesting that these three genes may serve as potential biomarkers for predicting POAF. This finding holds promise for subsequent studies aiming at preoperative prediction of POAF.

As pivotal components facilitating the coupling of amino acids with nucleotide triplets within tRNAs, aminoacyl-tRNA synthetases are posited to represent among the earliest proteins in evolutionary history. Aminoacyl-tRNA synthetases have two subtypes, and WARS2 is one of them. Wang’s seminal research [22] distinctly elucidates the integral role of WARS2 in cardiac vascular formation. Zebrafish experiments, involving the inhibition of WARS2, revealed compromised survival of extracardiac vessels and a concurrent disruption in the interaction between endocardium and myocardium. Similarly, suppressing WARS2 expression in rats resulted in impaired cardiac vascular generation and diminished myocardial capillary density. These findings unequivocally affirm WARS2's influential role in both intra- and extracardiac vascular development. While vascular regeneration is pivotal for cardiac repair and reperfusion, its dysregulation may induce structural and functional cardiac alterations, potentially elevating the risk of AF.

CKAP2 is a cytoskeleton-associated protein that stabilizes microtubules and plays a role in the regulation of cell division. The encoded protein is itself regulated through phosphorylation at multiple serine and threonine residues. Chen's study [23] revealed that the expression of CKAP2 in retinal capillary endothelial cells is regulated by the vascular endothelial growth factor and p53. The research findings indicate that the increased expression of CKAP2 is associated with cell proliferation, migration, and enhanced vascular formation. Wu [24] conducted Mendelian randomization analysis to explore genes associated with stroke in the blood, including CKAP2. Previous research has consistently shown that the occurrence of POAF is linked to an increased risk of future stroke in patients. It is worth investigating whether there is a relationship between the impact of POAF on stroke incidence and the presence of the CKAP2 gene.

CHI3L1 is a glycoprotein member of the chitinase 18 family, lacking chitinase activity. It is secreted by activated macrophages, chondrocytes, neutrophils, and synovial cells. The protein is believed to play a role in the processes of inflammation and tissue remodeling. Numerous recent studies suggest that inflammation plays a crucial role in the pathogenesis of POAF [25]. Through the analysis of the correlation between preoperative and postoperative neutrophil/lymphocyte levels and the incidence of POAF, it has been observed that an increase in the ratio is associated with an increased incidence of POAF [26]. Henry [27] utilized proteomics to assess the association between 90 cardiovascular proteins and heart failure events. Through Mendelian randomization, eight proteins were identified to consistently associate with heart failure. Among them was CHI3L1, and notably, it exhibited a negative correlation with heart failure. In this study, it was observed that CHI3L1 expression in POAF (+) patients was lower compared to POAF (-) patients, suggesting that decreased CHI3L1 expression may elevate the risk of heart failure in patients. This finding aligns with previous research indicating that the occurrence of POAF increases the risk of subsequent heart failure [28]. Therefore, further exploration is needed to determine whether CHI3L1 mediates the occurrence of POAF, thereby contributing to an increased risk of heart failure in patients.

In summary, the genes WARS2 and CKAP2 may be associated with vascular growth and migration, while CHI3L1 demonstrates a potential correlation with inflammation in patients. All three genes have the potential to serve as biomarkers for predicting POAF and could be explored as therapeutic targets in the management of POAF.

## Limitation

Our study had several limitations. First, there were only two mRNA datasets and one DNA methylation dataset available for POAF from CABG patients. These datasets were produced utilizing several platforms, and the variety of sample types resulted in bias. Second, it is essential to investigate the underlying mechanism linking the detected DEGs, DMGs, and POAF.

## Supplementary Information

ESM 1 (XLSX 9 kb)

ESM 2 (XLSX 39 kb)

## Abbreviations

AF: Atrial Fibrillation
POAF: Postoperative Atrial Fibrillation
CABG: Coronary Artery Bypass Grafting
LASSO: Least Absolute Shrinkage and Selection Operator
AUC: Area Under the Curve
BMI: Body Mass Index
Scr: Serum Creatinine
LVEF: Left Ventricular Ejection Fraction
DM: Diabetes Mellitus
GEO: Gene Expression Omnibus
miRNA: MicroRNA
circRNA: Circular RNA
SR: Sinus Rhythm
FC: Fold Change
DEG: Differentially Expressed Gene
DMG: DNA Methylation Gene
DMP: DNA Methylation Position
GO: Gene Ontology
RCAB: Right Coronary Artery Bypass
KEGG: Kyoto Encyclopedia of Genes and Genomes
Hb: Hemoglobin
RT-qPCR: Real-Time Quantitative Polymerase Chain Reaction
PBMC: Peripheral Blood Mononuclear Cell
ROC: Receiver Operating Characteristic
BP: Biological Process
CC: Cellular Component
MF: Molecular Function
WARS2: Tryptophanyl tRNA Synthetase 2
CKAP2: Cytoskeleton associated Protein 2
CHI3L1: Chitinase 3 like 1
HSD17B6: Hydroxysteroid 17-beta Dehydrogenase 6

## References

[1] Dobrev D, Aguilar M, Heijman J, Guichard JB, Nattel S. Postoperative atrial fibrillation: mechanisms, manifestations and management. Nat Rev Cardiol. 2019;16(7):417–36. 10.1038/s41569-019-0166-5. 30792496 10.1038/s41569-019-0166-5

[2] Rezk M, Taha A, Nielsen SJ, Gudbjartsson T, Bergfeldt L, Ahlsson A, et al. Clinical Course of Postoperative Atrial Fibrillation After Cardiac Surgery and Long-term Outcome. Ann Thorac Surg. 2022;114(6):2209–15. 10.1016/j.athoracsur.2022.03.062. 35430224 10.1016/j.athoracsur.2022.03.062

[3] Lin MH, Kamel H, Singer DE, Wu YL, Lee M, Ovbiagele B. Perioperative/Postoperative Atrial Fibrillation and Risk of Subsequent Stroke and/or Mortality. Stroke. 2019;50(6):1364–71. 10.1161/STROKEAHA.118.023921. 31043148 10.1161/STROKEAHA.118.023921

[4] Woldendorp K, Farag J, Khadra S, Black D, Robinson B, Bannon P. Postoperative Atrial Fibrillation After Cardiac Surgery: A Meta-Analysis. Ann Thorac Surg. 2021;112(6):2084–93. 10.1016/j.athoracsur.2020.10.055. 33340521 10.1016/j.athoracsur.2020.10.055

[5] Thoren E, Wernroth ML, Christersson C, Grinnemo KH, Jideus L, Stahle E. Compared with matched controls, patients with postoperative atrial fibrillation (POAF) have increased long-term AF after CABG, and POAF is further associated with increased ischemic stroke, heart failure and mortality even after adjustment for AF. Clin Res Cardiol. 2020;109(10):1232–42. 10.1007/s00392-020-01614-z. 32036429 10.1007/s00392-020-01614-z PMC7515855

[6] Gaudino M, Di Franco A, Rong LQ, Piccini J, Mack M. Postoperative atrial fibrillation: from mechanisms to treatment. Eur Heart J. 2023;44(12):1020–39. 10.1093/eurheartj/ehad019. 36721960 10.1093/eurheartj/ehad019 PMC10226752

[7] Peng Y, Su P, Zhao L. Long noncoding RNA and messenger RNA profiling in epicardial adipose tissue of patients with new-onset postoperative atrial fibrillation after coronary artery bypass grafting. Eur J Med Res. 2024;29(1):134. 10.1186/s40001-024-01721-x. 38368363 10.1186/s40001-024-01721-x PMC10874008

[8] Khan MS, Yamashita K, Sharma V, Ranjan R, Dosdall DJ. RNAs and Gene Expression Predicting Postoperative Atrial Fibrillation in Cardiac Surgery Patients Undergoing Coronary Artery Bypass Grafting. J Clin Med. 2020;9(4) 10.3390/jcm9041139. 10.3390/jcm9041139 PMC7231013 32316120

[9] Li XY, Hou HT, Chen HX, Liu XC, Wang J, Yang Q, et al. Preoperative plasma biomarkers associated with atrial fibrillation after coronary artery bypass surgery. J Thorac Cardiovasc Surg. 2021;162(3):851–63. 10.1016/j.jtcvs.2020.01.079. 32197906 10.1016/j.jtcvs.2020.01.079

[10] Skaria R, Parvaneh S, Zhou S, Kim J, Wanjiru S, Devers G, et al. Path to precision: prevention of post-operative atrial fibrillation. J Thorac Dis. 2020;12(5):2735–46. 10.21037/jtd-19-3875. 32642182 10.21037/jtd-19-3875 PMC7330352

[11] Qureshi M, Ahmed A, Massie V, Marshall E, Harky A. Determinants of atrial fibrillation after cardiac surgery. Rev Cardiovasc Med. 2021;22(2):329–41. 10.31083/j.rcm2202040. 34258901 10.31083/j.rcm2202040

[12] Bu D, Luo H, Huo P, Wang Z, Zhang S, He Z, et al. KOBAS-i: intelligent prioritization and exploratory visualization of biological functions for gene enrichment analysis. Nucleic Acids Res. 2021;49(W1):W317–W25. 10.1093/nar/gkab447. 34086934 10.1093/nar/gkab447 PMC8265193

[13] Bardou P, Mariette J, Escudié F, Djemiel C, Klopp C. jvenn: an interactive Venn diagram viewer. BMC Bioinformatics. 2014;15(1):293. 10.1186/1471-2105-15-293. 25176396 10.1186/1471-2105-15-293 PMC4261873

[14] Yiin GS, Howard DP, Paul NL, Li L, Mehta Z, Rothwell PM, et al. Recent time trends in incidence, outcome and premorbid treatment of atrial fibrillation-related stroke and other embolic vascular events: a population-based study. J Neurol Neurosurg Psychiatry. 2017;88(1):12–8. 10.1136/jnnp-2015-311947. 26487646 10.1136/jnnp-2015-311947 PMC5256147

[15] Eikelboom R, Sanjanwala R, Le ML, Yamashita MH, Arora RC. Postoperative Atrial Fibrillation After Cardiac Surgery: A Systematic Review and Meta-Analysis. Ann Thorac Surg. 2021;111(2):544–54. 10.1016/j.athoracsur.2020.05.104. 32687821 10.1016/j.athoracsur.2020.05.104

[16] Higuchi S, Kabeya Y, Matsushita K, Arai N, Tachibana K, Tanaka R, et al. Perioperative Atrial Fibrillation in Noncardiac Surgeries for Malignancies and One-Year Recurrence. Can J Cardiol. 2019;35(11):1449–56. 10.1016/j.cjca.2019.07.008. 31679617 10.1016/j.cjca.2019.07.008

[17] Mauro MD, Calafiore AM, Di Franco A, Nicolini F, Formica F, Scrofani R, et al. Association between cardioplegia and postoperative atrial fibrillation in coronary surgery. Int J Cardiol. 2021;324:38–43. 10.1016/j.ijcard.2020.09.065. 33022288 10.1016/j.ijcard.2020.09.065 PMC7874574

[18] Corradi D, Saffitz JE, Novelli D, Asimaki A, Simon C, Oldoni E, et al. Prospective Evaluation of Clinico-Pathological Predictors of Postoperative Atrial Fibrillation: An Ancillary Study From the OPERA Trial. Circ Arrhythm Electrophysiol. 2020;13(8):e008382. 10.1161/CIRCEP.120.008382. 32654517 10.1161/CIRCEP.120.008382 PMC7457312

[19] Kowalewski M, Pawliszak W, Raffa GM, Malvindi PG, Kowalkowska ME, Zaborowska K, et al. Safety and efficacy of miniaturized extracorporeal circulation when compared with off-pump and conventional coronary artery bypass grafting: evidence synthesis from a comprehensive Bayesian-framework network meta-analysis of 134 randomized controlled trials involving 22 778 patients. Eur J Cardiothorac Surg. 2016;49(5):1428–40. 10.1093/ejcts/ezv387. 26537755 10.1093/ejcts/ezv387

[20] Nagappa M, Ho G, Patra J, Wong J, Singh M, Kaw R, et al. Postoperative Outcomes in Obstructive Sleep Apnea Patients Undergoing Cardiac Surgery: A Systematic Review and Meta-analysis of Comparative Studies. Anesth Analg. 2017;125(6):2030–7. 10.1213/ANE.0000000000002558. 29049073 10.1213/ANE.0000000000002558

[21] Harling L, Lambert J, Ashrafian H, Darzi A, Gooderham NJ, Athanasiou T. Elevated serum microRNA 483-5p levels may predict patients at risk of post-operative atrial fibrillation. Eur J Cardiothorac Surg. 2017;51(1):73–8. 10.1093/ejcts/ezw245. 27422887 10.1093/ejcts/ezw245 PMC5226070

[22] Wang M, Sips P, Khin E, Rotival M, Sun X, Ahmed R, et al. Wars2 is a determinant of angiogenesis. Nat Commun. 2016;7:12061. 10.1038/ncomms12061. 27389904 10.1038/ncomms12061 PMC4941120

[23] Chen X, Xie J, Cui Y, Zhang L, Yu H, Chen J, et al. Cytoskeleton-associated protein 2 (CKAP2) is regulated by vascular endothelial growth factor and p53 in retinal capillary endothelial cells under high-glucose conditions. Mol Cell Endocrinol. 2021;535:111378. 10.1016/j.mce.2021.111378. 34216644 10.1016/j.mce.2021.111378

[24] Wu BS, Chen SF, Huang SY, Ou YN, Deng YT, Chen SD, et al. Identifying causal genes for stroke via integrating the proteome and transcriptome from brain and blood. J Transl Med. 2022;20(1):181. 10.1186/s12967-022-03377-9. 35449099 10.1186/s12967-022-03377-9 PMC9022281

[25] Zakkar M, Ascione R, James AF, Angelini GD, Suleiman MS. Inflammation, oxidative stress and postoperative atrial fibrillation in cardiac surgery. Pharmacol Ther. 2015;154:13–20. 10.1016/j.pharmthera.2015.06.009. 26116810 10.1016/j.pharmthera.2015.06.009

[26] Gibson PH, Cuthbertson BH, Croal BL, Rae D, El-Shafei H, Gibson G, et al. Usefulness of neutrophil/lymphocyte ratio as predictor of new-onset atrial fibrillation after coronary artery bypass grafting. Am J Cardiol. 2010;105(2):186–91. 10.1016/j.amjcard.2009.09.007. 20102916 10.1016/j.amjcard.2009.09.007

[27] Henry A, Gordillo-Marañón M, Finan C, Schmidt AF, Ferreira JP, Karra R, et al. Therapeutic Targets for Heart Failure Identified Using Proteomics and Mendelian Randomization. Circulation. 2022;145(16):1205–17. 10.1161/circulationaha.121.056663. 35300523 10.1161/CIRCULATIONAHA.121.056663 PMC9010023

[28] Goyal P, Kim M, Krishnan U, McCullough SA, Cheung JW, Kim LK, et al. Post-operative atrial fibrillation and risk of heart failure hospitalization. Eur Heart J. 2022;43(31):2971–80. 10.1093/eurheartj/ehac285. 35764099 10.1093/eurheartj/ehac285 PMC9890619

